# Safety, Tolerability, and Pharmacokinetics of NorUrsodeoxycholic Acid (Shilpa Medicare Limited, India) in Healthy Adults: Results From a Phase I Open-Label Dose-Escalation Study

**DOI:** 10.1155/adpp/6613969

**Published:** 2025-07-17

**Authors:** Veerendra Kumar Panuganti, Chandrasekhar Varma Alluri, Mamatha Reddy Dundigalla, Javeed Mohammad, Pavan Kumar Madala, Sanyasirao K. S. S. V. V.

**Affiliations:** ^1^Clinical Affairs, Shilpa Medicare Ltd, Hyderabad, Telangana, India; ^2^Medical Affairs Department, Shilpa Medicare Ltd, Hyderabad, Telangana, India; ^3^Bioanalytical Laboratory, Shilpa Medicare Ltd, Hyderabad, Telangana, India

**Keywords:** 24-norUrsodeoxycholic acid, dose-limiting toxicity, healthy subjects, maximum tolerated dose, pharmacokinetic, phase I clinical trials, safety

## Abstract

NorUrsodeoxycholic acid (norUDCA) is a side-chain-shortened derivative of ursodeoxycholic acid (UDCA) with specific pharmacological properties, including cholehepatic shunting, making it a promising candidate for a range of cholestatic and metabolic liver diseases. This phase I study evaluates the safety, tolerability, and pharmacokinetics of single ascending doses of norUDCA in healthy adults under fasting conditions. In this open-label, dose-escalation study, healthy adults (aged 18–45 years) were enrolled into 3 successive dose escalation cohorts, receiving norUDCA oral tablets of 500 mg (*n* = 14), 1000 mg (*n* = 14), and 1500 mg (*n* = 14) once daily. The primary endpoints were the incidence rate of dose-limiting toxicities (DLTs), assessment of maximum tolerated dose (MTD) after single dose administration, and an establishment of the recommended phase II dose. Secondary endpoints included pharmacokinetic assessments and evaluation of dose proportionality. Following single-dose administration of 500 mg, 1000 mg, and 1500 mg, no DLTs were observed, and the MTD was determined to be 1500 mg/day. Maximum plasma concentration (*C*_max_) and plasma exposure [area under the curve (AUC)] to norUDCA increased dose proportionally from 500 to 1500 mg/day. Median time to achieve maximum plasma concentration (*T*_max_) for norUDCA at 1500 mg/day cohort (3 h) was comparable to 1000 mg/day cohort (3 h) but lower than 500 mg/day cohort (4 h). The half-life was longer in 1500 mg/day (16 h) cohort compared to 500 mg/day (15 h) and 1000 mg/day cohorts (14 h). The 90% confidence interval of the slope estimates for *C*_max_ (22.41, 59.70), AUC_0−*t*_ (11.71, 61.14), and AUC_0−inf_ (10.74, 61.86) did not consistently fall within the predefined acceptable limits (79.69, 120.31) for formal dose proportionality criteria. No serious adverse events or deaths occurred in any cohort. One treatment-emergent adverse event was observed (an increase in the white blood cell count), which was not related to the study drug. A single dose of norUDCA is safe and well-tolerated, with favorable plasma pharmacokinetic profiles in healthy subjects. Based on the safety and pharmacokinetic data, the recommended dosage for further clinical trials is 1500 mg/day.

**Trial Registration:** Clinical Trials Registry-India: CTRI/2022/11/047561

## 1. Introduction

The new derivative of ursodeoxycholic acid (UDCA), 24-norUrsodeoxycholic acid (norUDCA), is a side-chain shortened C23 homolog of UDCA with relative resistance to side chain conjugation (amidation with glycine and taurine). Unlike UDCA, norUDCA is a poor substrate for acyl-coenzyme A synthetase and undergoes only minimal N-acyl amidation with taurine or glycine, which leads to cholehepatic shunting [1], resulting in hepatic enrichment [1, 2]. This enrichment enables norUDCA to directly exert therapeutic effects on both parenchymal and nonparenchymal liver cells, thereby alleviating cholestasis, steatosis, hepatic inflammation, and fibrosis, reducing hepatocellular proliferation, and promoting autophagy [3]. This pharmacological properties may pave the way for therapeutic application of norUDCA in a broad range of cholestatic conditions (e.g., primary biliary cholangitis, primary sclerosing cholangitis (PSC), cystic fibrosis, sclerosing cholangitis in critically ill patients, and ABCB4 deficiency such as progressive familial intrahepatic cholestasis, low phospholipid-associated cholelithiasis, and cholemic nephropathy) and metabolic conditions (e.g., nonalcoholic fatty liver disease (NAFLD)/nonalcoholic steatohepatitis (NASH), atherosclerosis, and alpha-1 antitrypsin (A1AT) deficiency) [3, 4]).

Despite its extensive therapeutic potential, there is limited clinical evidence supporting the therapeutic benefits of norUDCA. Only two clinical trials have demonstrated its effectiveness in treatment of PSC (phase II; *n* = 161) [5] and NAFLD/NASH (phase II, *n* = 198) [6]. Moreover, norUDCA has shown a favorable safety profile comparable to placebo up to a dose of 1500 mg daily during the 12-week treatment period in diseased patients [5, 6]. However, there is still a lack of data regarding the safety and pharmacokinetic (PK) of norUDCA.

Shilpa Medicare Limited, India, has developed a new norUDCA molecule, which has shown good safety and tolerability in preclinical studies performed under fasting conditions (water was allowed, except restricted from at least 1 hour prior to dosing), as well as potentially enhanced efficacy when used at a higher dose (data on the file). The current phase II study is designed to evaluate the safety and tolerability of single ascending doses of norUDCA (500 mg/kg, 1000 mg/kg, and 1500 mg/kg daily) in healthy adult human subjects under fasting conditions and to establish the recommended doses for future clinical studies. An additional goal is to characterize the single-dose oral PK profile of norUDCA under fasting conditions.

## 2. Method

### 2.1. Study Design and Study Population

This was an open-label, single-center, dose-escalation phase I study designed to determine the safety, tolerability, and PK of norUDCA given orally to healthy adult Indian subjects (CTRI/2022/11/047561). A total of 42 subjects were included in the study, with 12 subjects in each of three cohorts (500 mg, 1000 mg, and 1500 mg). Eligibility criteria included males and/or nonpregnant, nonlactating female subjects of age 18–45 years (both inclusive) with a body mass index (BMI) of 18.5–30.0 kg/m^2^. Subjects were enrolled into the study upon the acceptable screening evaluations, which included demographics, physical examination including vital signs, past medical and medication history, 12-lead ECG, urine drug screening, serum pregnancy test for female subjects with child-bearing potential, chest x-ray, and routine laboratory tests (i.e., hematology, biochemistry, urinalysis, and serology).

Main exclusion criteria were a history or presence of hypersensitivity, diabetes, bronchial asthma, psychosis, significant diseases, difficulty in swallowing, or any gastrointestinal disease that could affect drug absorption. Healthy subjects who underwent hormone replacement therapy within the last 3 months, or received a depot injection/implant of any drug within the 3 months, or received cytochrome P enzyme inhibitors/inducers within the last 30 days prior to the administration of study drug, as well as received any prescribed medications within the last 14 days, or any over the counter (OTC) products, vitamins, or herbal products within the last 7 days prior to housing, were excluded. Healthy subjects who used grapefruit and grapefruit-containing products within 7 days or ingestion of any caffeine or xanthine products (i.e., coffee, tea, chocolate, caffeine-containing sodas, and colas), cigarettes and tobacco containing products, recreational drugs, or alcohol/alcohol-containing products within 96 h prior to housing were also excluded. Healthy subjects were excluded if they had taken a known investigational drug within seven elimination half-lives (*t*_1/2_) of the administered study drug prior to the housing, had a history or evidence of drug dependence or alcoholism, or moderate alcohol use, smokers (10 or more cigarettes per day or 20 or more biddies per day), or those who could not refrain from smoking during the study period. Additionally, those with a history of difficulty in donating blood or accessibility of veins tested positive for hepatitis B and C, HIV antibody, and/or syphilis, had donated or lost 50–100 mL of blood within 30 days or 101–200 mL within 60 days or > 200 mL within 90 days (excluding volume drawn at screening for this study) prior to dosing of study drug whichever greater, and were excluded. Subjects with intolerance to venipuncture had any food allergy, intolerance, restriction, or special diet that, in the opinion of the principal investigator or subinvestigator, could contraindicate their participation in this study, or those who had ingested any unusual diet, for any reason (e.g., low sodium), for 3 weeks prior to housing, were excluded.

The study protocol, amendments, and informed consent form were reviewed and approved by the Institutional Ethics Committee (IEC). All subjects provided written informed consent prior to the commencement of any study-specific procedures. The study was conducted in accordance with pertinent requirements of the Declaration of Helsinki (ethical principles for medical research involving human subjects, revised by the 64th World Medical Association (WMA) General Assembly, Brazil, October 2013), relevant to the United States Food and Drug Administration (USFDA) guidelines and the International Council for Harmonisation (ICH) E6–Good Clinical Practice (GCP) (R2) guidelines, along with the local regulatory requirements of GCP for clinical research In India [2004, Central Drugs Standard Control Organization (CDSCO)], New Drugs and Clinical Trials Rules, 2019, the Indian Council of Medical Research's (ICMR), and National Ethical guidelines for Biomedical and Health Research involving Human Participants (2017).

### 2.2. Dose Escalation

**Figure 1:**
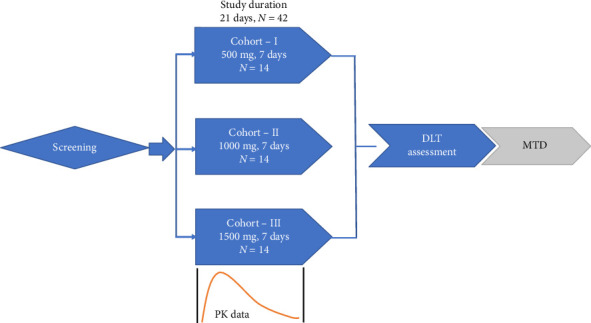
Study cohorts.

Subjects were to take 500 mg tablets of norUDCA for oral use. Subjects were randomly assigned to receive single ascending doses of norUDCA tablets at 500 mg per day or 1000 mg per day (2 × 500 mg) or 1500 mg (3 × 500 mg) per day. The study duration for each cohort was 7 days from the day of check-in (housing in the clinic at least 60 h prior to dosing until at least 72 h postdose) (Figure 1). norUDCA tablets were administered orally in a sitting posture with about 240 mL of water at ambient temperature under the supervision of trained study personnel following a fasting for at least 10 hours prior to dosing and water restriction from at least 1 hour prior to dosing. Fasting was continued until at least 4 hours postdose, with water restriction for at least 1 hour postdose (no fluid, except water was given with dosing). The study was conducted under fasting conditions to minimize variability in absorption and metabolism that can occur due to food intake and also to isolate the drug's effects and interactions within the body. Subjects were instructed to remain in sitting posture for initial 4 hours after dosing, with only necessary movement allowed during this period. This position was maintained to ensure uniform and complete absorption of norUDCA in all subjects and to decrease the potential risk of gastroesophageal adverse events (AEs) due to norUDCA.

Based on the published literature, the starting first adult human dose was 500 mg/day. Subsequent dose escalations of 1000 mg/day and 1500 mg/day would be proposed based on a 100% dose escalation and maximum dose received being 1500 mg/day [5, 6]. The dose escalation was performed in cohorts of 14 subjects. Decisions to escalate or stop followed the steps as defined in stopping criteria.

#### 2.2.1. Stopping Criteria

If no dose-limiting toxicities (DLTs) were observed in any of these subjects, next dose as per protocol was administered. The next highest dose cohort was not dosed for at least 3 days following the discharge of the last subject from the preceding cohort. If one serious adverse reaction (DLT) or more than six out of the 14 subjects experienced severe nonserious adverse reaction (DLTs), then the previous dose level was considered as maximum tolerated dose (MTD). The MTD was the highest dose at which one serious adverse reaction (DLTs) or more than six of 14 subjects experience DLTs. Since MTD was not reached even with the highest dose level of 1500 mg/day, the study was stopped, and there was no further dose escalation. All DLTs were considered when making decisions regarding drug escalation increments or discontinuations, unless they are clearly related to something other than the investigational product.

Subjects were observed for DLTs for 72 h after single dose administration of study drug at each dose level. The subjects were observed for the following predefined and listed DLTs in the study: Hematological toxicities: creatinine increased (Grade 3: > 3.0–6.0 × upper limit of normal (ULN), Grade 4: > 6.0 × ULN, and Grade 3 leukopenia. Nonhematological toxicities: Grade ≥ 3 toxicities excluding Grade 3 nausea and vomiting resolving to Grade ≤ 1 within 72 h, with or without adequate antiemetic treatment, Grade 3 pruritus, Grade 3 diarrhea that resolves to Grade ≤ 1 within 72 h, Grade 3 fatigue that resolves to Grade ≤ 1 within 72 h, Grade 3 seizures, and any death [7, 8].

### 2.3. Study Endpoints and Assessments

The primary endpoints were the incidence rate of drug-related DLTs (safety) and assessment of MTD (tolerability) after single-dose administration at dose levels of 500 mg/day, 1000 mg/day, and 1500 mg/day, as well as establishing the recommended phase II dose(s) and regimen of norUDCA. Secondary endpoints include characterization of primary PK parameters [maximum serum concentration (*C*_max_), the area under the curve from time zero to *t* (AUC_0−*t*_), and the area under the curve from time zero extrapolated to infinity (AUC_0−inf_)]. Secondary PK parameters included time to achieve maximum serum concentration (*T*_max_), elimination rate constant (*K*_el_), *t*_1/2_, clearance of drug observed (Cl_F_obs), volume of distribution of the drug observed (Vd_F_obs), and percentage of AUC that has been derived after extrapolation (AUC_% Extrap_Obs). Additionally, dose proportionality was assessed after single dose administration of norUDCA at dose levels of 500 mg/day, 1000 mg/day, or 1500 mg/day. The primary PK parameters (*C*_max_, AUC_0−*t*_, and AUC_0−inf_) were used to calculate dose proportionality.

#### 2.3.1. Safety Assessment

Safety assessment included monitoring of AEs and serious AEs (SAEs), clinical laboratory test results, vital sign measurements (blood pressure, pulse rate, respiratory rate, and body temperature), 12-lead electrocardiogram, chest x-ray, physical examination findings, urine screen for drugs of abuse, and urine alcohol test.

#### 2.3.2. PK Assessment

Blood samples for determination of norUDCA were collected at the following time points after single dose for each dose level: baseline (−48.0, −42.0, −36.0, −30.0, −24.0, −18.0, −12.0, and −6.00), predose (0.0) prior to dosing within 2 h, and post dose within +2 min (0.083, 0.167, 0.333, 0.5, 0.667, 0.833, 1.0, 1.167, 1.334, 1.5, 1.667, 1.834, 2.0, 2.5, 3.0, 4.0, 6.0, 8.0, 10.0, 12.0, 16.0, 24.0, 36.0, 48.0, and 72.0 h). Plasma samples were analyzed by Bioanalytical Lab, Shilpa Medicare Limited, using a validated liquid chromatography-mass spectrometry (LCMS/MS) method.

### 2.4. Statistical Method

The sample size chosen for this study was selected without statistical considerations. We consider the sample size defined for Phase I study to be adequate to meet the study objectives.

No formal hypothesis testing was conducted; all statistical analyses for demographic, safety, and PK were summarized using descriptive statistics by dose cohort. Dose proportionality of baseline-corrected *C*_max_, AUC_0−*t*_, and AUC_0−inf_ values for norUDCA was assessed graphically and with the standard power model. An analysis of variance (ANOVA) method was performed on the ln-transformed PK parameter: baseline-corrected *C*_max_, AUC_0−*t*_, and AUC_0−inf_.

#### 2.4.1. Criteria for Dose Proportionality

Dose proportionality was evaluated graphically and using the power model as described below:

(1) $$\begin{array}{c} \log_{e}\left(\text{PK parameter}\right)=a+b\times \log_{e}\left(\text{dose}\right), \end{array}$$

where *a* = intercept and *b* = slope.

The critical interval was calculated as follows:

The ratio (*r*) of the highest dose level to the lowest dose level was calculated. The lower limit of the critical interval was calculated as follows:

(2) $$\begin{array}{c} \frac{\log_{e}\left(0.8\right)}{\log_{e}\left(r\right)}+1. \end{array}$$

The upper limit of the critical interval was calculated as follows:

(3) $$\begin{array}{c} \frac{\log_{e}\left(1.25\right)}{\log_{e}\left(r\right)}+1. \end{array}$$

The *r* value is based on the ratio between the highest and lowest dose levels. For this single dose, the planned dose level range was 500–1500 mg unit of norUDCA, and *r* equals 1500/500 = 3. Thus, the critical interval for planned dose ranges to declare dose linearity was 0.7969–1.2031 for the 90% confidence interval (CI) of the slope estimate. The critical interval was estimated for actual dose ranges used in the study.

PK parameters of norUDCA were calculated by the noncompartmental model by Phoenix WinNonlin software version 8.1.1 or higher (Pharsight Corporation, USA). All below limit of quantitation (BLQ) concentration values were set to zero before PK analysis. All statistical analysis was performed using SAS version 9.4 or higher (SAS Institute Inc, Cary, NC, USA). The norUDCA concentration-time data were presented using the linear and semilogarithmic transformed plot.

## 3. Results

Between 8th November 2022 and 23rd December 2022, 42 healthy subjects [median age (range): 33.0 (18–43) years; predominantly male (66.67%)] were enrolled in this dose-escalation study. The subjects were randomized into three norUDCA dose cohorts: 500 mg/day, 1000 mg/day, and 1500 mg/day, with 14 subjects in each cohort.  Table 1 outlines the demographic and baseline data for each cohort.

Following single-dose administration of 500 mg, 1000 mg, and 1500 mg, no DLTs were observed, and the MTD was determined to be 1500 mg/day.

**Figure 2:**
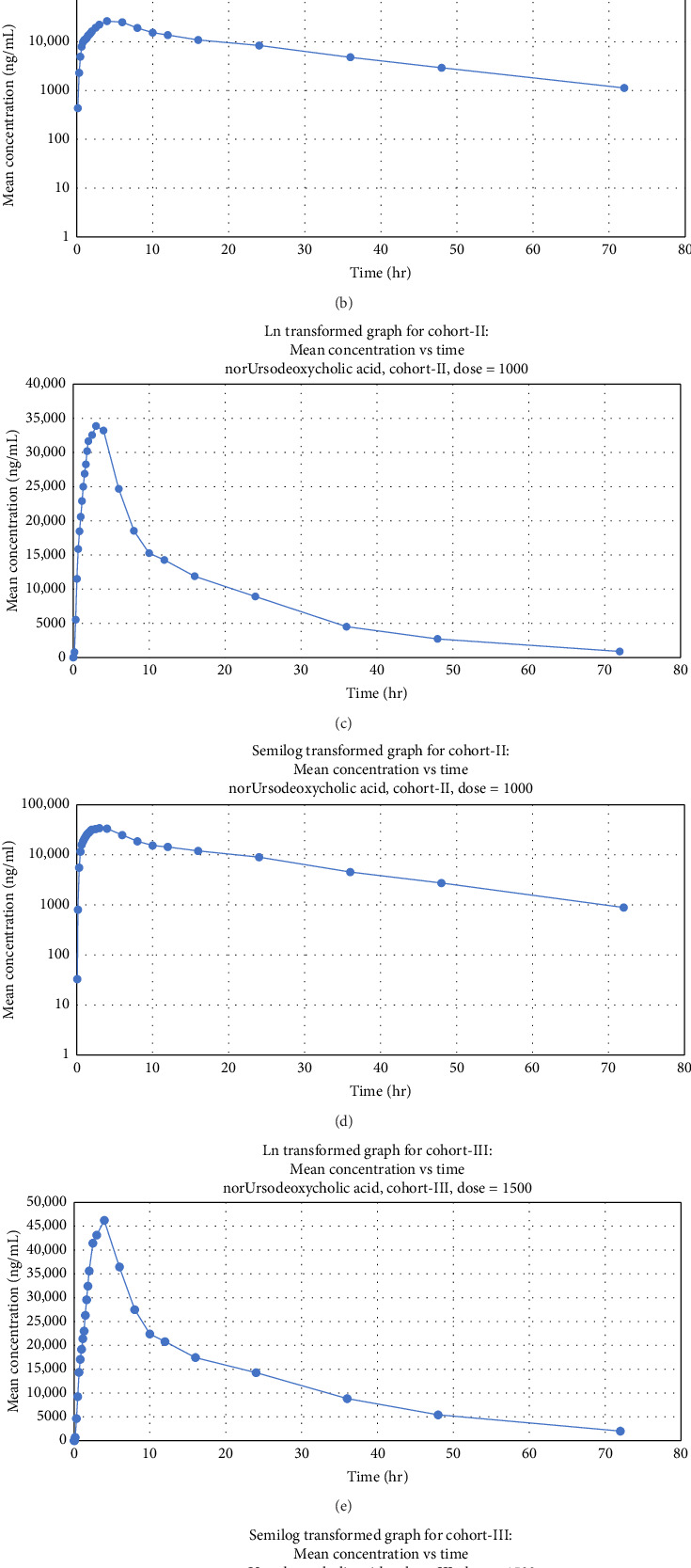
Mean plasma concentration of norUrsodeoxycholic acid versus time after a single dose. Cohort 1 (500 mg) (a) natural logarithm (ln) transformed scales, (b) semilog transformed scale; cohort 2 (1000 mg), (c) natural logarithm (ln) transformed linear scale, (d) semilog transformed scale; cohort 3 (1500 mg) (e) natural logarithm (ln) transformed scales, (f) semilog transformed scale.

The PK data of single dose norUDCA at the dose level of 500 mg/day, 1000 mg/day, and 1500 mg/day are presented in Table 2. *C*_max_ of norUDCA increased in a dose-proportional manner in the dose range of 500 mg/day to 1500 mg/day in the single-dose phase. Similarly, AUC values of norUDCA also increased in dose-proportional manner. *T*_max_ of norUDCA at 1500 mg/day (3 h) was similar to that at 1000 mg/day (3 h) but shorter than that at 500 mg/day (4 h). Moreover, *t*_1/2_ of norUDCA in the 1500 mg/day cohort was longer (16 h) compared to 500 mg/day (15 h) and 1000 mg/day cohorts (14 h). Plasma-concentration time profiles under fasting condition showed similar patterns of exposure that appeared to be dose-dependent (Figure 2).

In the analysis of dose proportionality using the power model, the slope estimates were 41.05 (90% CI: 22.41, 59.70) for *C*_max_, 36.42 (90% CI: 11.71, 61.14) for AUC_0−*t*_, and 36.30 (90% CI: 10.74, 61.86) for AUC_0−inf_. All of these 90% CIs of the slope estimates did not consistently fall within the predefined acceptable limits (79.69, 120.31) for formal dose proportionality criteria (Table 3).

One treatment emergent AE was experienced by one (1/14 = 7.14%) subject in Cohort 3 (1500 mg); the reported AE was an increase in the white blood cell count (Table 4). The event was characterized as mild in severity and unlikely to be related to study drug. No subjects were withdrawn from the study, and no SAEs or deaths occurred during the entire study period.

## 4. Discussion

To our knowledge, this is the first study to evaluate the safety, tolerability, and PK of norUDCA in healthy adult human subjects following a single oral dose administration. This phase I study demonstrates that norUDCA is safe and well-tolerated at dose levels of 500 mg/day, 1000 mg/day, and 1500 mg/day, with favorable plasma PK profiles in healthy subjects. No DLTs were reported with single doses of norUDCA ranging from 500 to 1500 mg daily under fasting condition. The MTD was established at 1500 mg per day.

We have conducted three comprehensive preclinical toxicity studies to assess the safety and potential toxicity of this newly formulated compound, norUDCA. The first two studies aimed to identify target organ toxicity and establish the no observed effect level (NOEL)/no observed adverse effect level (NOAEL) for norUDCA. In these repeat-dose oral gavage toxicity studies, norUDCA was administered orally at doses of 1250 mg/kg, 2500 mg/kg, and 5000 mg/kg to Wistar rats and Swiss albino mice for 28 weeks. Both the studies have proven that norUDCA blend up to highest dose (5000 mg/kg/day) did not reveal any adverse toxicological effects. The NOAEL of norUDCA was considered to be 5000 mg/kg/day for Wistar rats and Swiss albino mice (Institutional Animal Ethic Committee (IAEC) approval no. LBPL-IAEC-054-04/2020). The third study was a dose-range finding study, focused on determining MTD of norUDCA for subsequent studies. In this study, norUDCA (1000 mg/kg, 2000 mg/kg, 3000 mg/kg, and 5000 mg/kg) was administered orally in Wistar rats over 10 days. An MTD of 5000 mg/kg/day was established with repeat norUDCA dosing for Wistar rats (IAEC approval no. LBPL-IAEC-010-01/2020).

We evaluated the effect of the investigational product (norUDCA) in a Gubra Amylin diet plus CCl_4_-induced NASH model in male C57BL6 mice. From the result of this efficacy study, the action of norUDCA against NAFLD has been documented. It was found that norUDCA decreases the expression of transforming growth factor beta (TGF-*β*) and collagen type I and stimulates the farnesoid X receptor (FXR). The FXR regulates the synthesis and transport of bile acids and maintains its homeostasis. The FXR also influences the hepatic lipid and glucose metabolism by regulating the cholesterol levels and increasing insulin sensitivity. The FXR further inhibits the proinflammatory cytokine production. TGF-β acts as a key driver of fibrosis, leading to excessive extracellular matrix deposition and tissue scarring, while collagen type I receptors, such as integrins, facilitate cell adhesion and migration, which are essential for tissue integrity and repair and play a vital role in the development of fibrosis by contributing to the excessive accumulation of extracellular matrix components, resulting in tissue scarring. By acting on these pathways, norUDCA decreases the fat content, inflammation, and fibrosis in the liver, thereby reversing the pathology of NAFLD.

Plasma concentrations of norUDCA reached their maximal level approximately 3-4 h after a single dose administration and then declined slowly with an apparent terminal *t*_1/2_ of approximately 14–16 h. Median *T*_max_ values for norUDCA at 1500 mg/day (3 h) were comparable to 1000 mg/day (3 h) cohort but lower than 500 mg/day (4 h) cohort. norUDCA undergoes cholehepatic circulation and has been reported to exhibit nonlinear PK. An increase in *t*_1/2_ and a decrease in clearance (*K*_el_) are expected with higher doses— this trend is observed between 1000 mg/day and 1500 mg/day cohorts. However, upon comparing 500 mg/day and 1000 mg/day cohorts, the clearance value appears higher in the 1000 mg/day cohorts. This could be explained by the fact that one subject in 500 mg/day cohort exhibited an aberrant high *T*_1/2_ value, leading to elevated mean values in that group compared to 1000 mg/day cohort. In the 1500 mg daily dose cohort, the observed *K*_el_ was lower (i.e., implying a slower rate of drug elimination) than in 500 mg/day and 1000 mg/day cohorts. Additionally, *t*_1/2_ was slightly longer in 1500 mg/day cohort compared to 500 mg and 1000 mg daily dose cohorts. The lower *K*_el_ and longer *t*_1/2_ suggest that norUDCA persists in the system for a longer duration at this dose, potentially reducing the need for frequent dosing to maintain desired exposures and high peak concentrations.

We conducted plasma sampling for up to 72 h postdose to cover both the drug release and postabsorptive elimination phases of norUDCA. The observed plasma profile with concentrations of 500, 1000, and 1500 mg, maintained over 72 h, supports the suggested regimen of administering norUDCA once daily.

Findings from the current study revealed a good oral absorption and systemic exposure of norUDCA with a single oral dose administration, across the range of dosing from 500 mg/day to 1500 mg/day; exposure with increasing dose was indicative of dose proportionality. Possible reasons for the increased exposure at higher doses include saturation of capacity of a metabolic route, autoinhibition of metabolism, or saturation of an efflux mechanism [9]. However, formal testing of dose proportionality was inconclusive, suggesting that norUDCA exhibit nonlinear PK across the dose range of 500 mg/day to 1500 mg/day. The lack of dose proportionality, implying nonlinear PK, may be due to various mechanisms such as saturation of carrier-mediated uptake, poor aqueous solubility or slow release from the formulation, and saturation of presystemic metabolism [10]. Lack of dose proportionality may have implications related to safety and efficacy [11]; however, in the current study, no safety concerns were observed in all three dose cohorts.

In the present study, there were no SAEs, deaths, or withdrawals occurred. One AE was reported by one subject, which was an increase in the white blood cell count. This event was of mild severity and not related to study drug. The safety profile of single dose norUDCA (500 mg/day, 1000 mg/day, and 1500 mg/day) observed in healthy adults in the present study is very encouraging. Consistent with these findings, two clinical trials with disease populations showed a favorable safety profile of norUDCA comparable to placebo [5, 6].

Although norUDCA demonstrates considerable theoretical therapeutic potential, only limited clinical evidence supports its efficacy in the treatment of PSC and NAFLD/NASH [5, 6]. To date, no pharmacological therapy has been approved by regulatory authorities for the treatment of either condition. UDCA is commonly used off-label for the treatment of PSC and NAFLD/NASH; however, its efficacy remains controversial. Several studies showed biochemical or histological improvements with UDCA in patients with PSC but failed to demonstrate significant effect on clinical outcomes, such as mortality, transplant-free survival, or development of cholangiocarcinoma [12–14]. Moreover, a study investigating the effects of UDCA withdrawal in patients with PSC demonstrated a significant deterioration of liver biochemistry and symptoms within 3 months [15]. Some clinical evidence showed that treatment with UDCA led to significant improvement in liver steatosis and lobular inflammation, as well as a notable reduction in serum aminotransferase levels and fibrosis markers in patients with NASH [16, 17]. However, other randomized controlled trials (RCTs) failed to demonstrate benefits of UDCA in attenuating steatosis and inflammation in patients with NASH [18]. Collectively, effective pharmacological therapy for both PSC and NAFLD/NASH is still an unmet clinical need, and novel drugs are urgently needed. norUDCA, a derivative of UDCA, holds promise as a potential alternative treatment for these conditions; however, further research is warranted to establish its clinical efficacy.

The results presented in the current study should be interpreted in light of certain limitations. First, it is a relatively small-sample size study. Second, the study was conducted in healthy adults using a parallel study design; patients might exhibit different PK properties due to factors such as disease progression or concomitant drugs. Although crossover study design is often adopted in PK studies to reduce the effect of interindividual variability, parallel study designs are also commonly used. Second, the PK assessment was conducted only under fasting conditions; hence, the impact of food on the PK profile of norUDCA could not be ascertained. Lastly, age and gender are significant factors affecting drug PK. The median age of our study cohorts was 33 years, and gender distribution varied significantly across the dose cohorts. Further evaluation involving a broader age range, including elderly subjects, a larger sample size, and balanced gender distribution, would be beneficial for determining the comprehensive PK characteristics of norUDCA. The current study has confirmed the favorable PK profile and safety of norUDCA. Based on these findings and the available literature, an appropriate dose of norUDCA could be selected for further studies. Further investigations of norUDCA in hepatic conditions such as NAFLD, PSC, and other cholestatic diseases may be planned.

## 5. Conclusions

NorUDCA was safe and well tolerated in healthy adult human subjects in this phase 1 dose escalation study. NorUDCA has a favorable safety and PK profile that supports further clinical studies. Based on the safety and PK data, the recommended dosage for future clinical trials is 1500 mg/day.

## Figures and Tables

**Table 1:** Demographic and baseline data (safety population).

Variables	Cohort 1 500 mg/day (*n* = 14)	Cohort 2 1000 mg/day (*n* = 14)	Cohort 3 1500 mg/day (*n* = 14)	Total subjects (*n* = 42)
Age (y)				
Median (min, max)	34.5 (22, 41)	36.0 (22, 43)	26.0 (18, 38)	33.0 (18, 43)
Sex, *n* (%)				
Male	0	14 (100)	14 (100)	28 (66.67)
Female	14 (100)	0	0	14 (33.33)
Race, *n* (%)				
Asian	14 (100)	14 (100)	14 (100)	42 (100)
Ethnicity, *n* (%)				
Not hispanic/latino	14 (100)	14 (100)	14 (100)	42 (100)
BMI (kg/m^2^)				
Median (min, max)	24.15 (21.3, 29.3)	23.30 (20.2, 28.7)	20.90 (18.7, 29.4)	23.05 (18.7, 29.4)
Height (cm)				
Median (min, max)	152.25 (139.0, 162.0)	164.50 (154.5, 178.0)	165.50 (155.5, 176.5)	162.00 (139.0, 178.0)
Weight (kg)				
Median (min, max)	54.60 (50.2, 73.2)	62.50 (53.2, 87.2)	56.00 (50.5, 84.0)	57.20 (50.2, 87.2)

Abbreviation: BMI = body mass index.

**Table 2:** Pharmacokinetic data of single dose of norUrsodeoxycholic acid (pharmacokinetics population).

Variables	Cohort 1 500 mg/day (*n* = 14)	Cohort 2 1000 mg/day (*n* = 14)	Cohort 3 1500 mg/day (*n* = 14)
*C*_max_ (ng/mL)			
Mean (±SD)	30,227.873 ± 6373.063	38,532.495 ± 14,305.046	49,945.629 ± 17,604.785
Geometric mean (% CoV)	29,521.916 (21.083)	36,221.110 (37.125)	47,061.476 (35.248)
AUC_0−*t*_ (ng/mL) ∗ (hr)			
Mean (±SD)	523,828.825 ± 136,381.350	570,073.700 ± 249,428.333	876,395.925 ± 389,701.864
Geometric mean (% CoV)	507,549.584 (26.035)	517,179.167 (43.754)	791,890.384 (44.466)
AUC_0−inf_ (ng/mL) ∗ (hr)			
Mean (±SD)	555,196.729 ± 158,119.276	592,115.430 ± 267,228.737	928,204.540 ± 415,159.359
Geometric mean (% CoV)	534,673.491 (28.480)	534,634.525 (45.131)	835,999.038 (44.727)
*T*_max_ (hr)			
Median (min, max)	4.000 (3.000, 6.000)	3.000 (1.334, 4.000)	3.000 (2.000, 4.000)
*K*_el_ (1/hr)			
Median (min, max)	0.045 (0.023, 0.063)	0.048 (0.033, 0.077)	0.043 (0.029, 0.084)
*t*_1/2_ (hr)			
Median (min, max)	15.493 (11.044, 29.835)	14.367 (9.051, 20.769)	16.070 (8.244, 23.802)
Cl_F_obs (mL/hr)			
Mean (±SD)	971.045 ± 276.929	2094.044 ± 1091.532	2010.564 ± 1022.004
Geometric mean (% CoV)	935.150 (28.519)	1870.437 (52.126)	1794.260 (50.832)
Vd_F_obs (mL)			
Mean (±SD)	22,335.291 ± 5735.463	38,696.634 ± 14,049.380	44,230.577 ± 19,954.844
Geometric mean (% CoV)	21,674.562 (25.679)	36,362.101 (36.306)	40,291.705 (45.115)
AUC_Extrap_obs (%)			
Mean (±SD)	4.990 ± 3.975	3.239 ± 2.326	5.231 ± 2.996
Geometric mean (% CoV)	3.919 (79.651)	2.549 (71.815)	4.476 (57.268)

*Note:C*_max_, maximum serum concentration; AUC_0−*t*_, area under the curve from time zero to *t*; AUC_0−inf_, area under the curve from time zero extrapolated to infinity; *T*_max_, time to achieve maximum serum concentration; *K*_el_, elimination rate constant; *t*_1/2_, half-life; Cl_F_obs, oral clearance of drug observed; Vd_F_obs, volume of distribution of drug observed; AUC_Extrap_obs, % of the AUC that has been derived after extrapolation.

Abbreviations: CoV = coefficient of variation, SD = standard deviation.

**Table 3:** Slope estimates, ratio, 90% confidence intervals, acceptance criteria, and outcomes of dose proportionality result based on ln-transformed data for norUrsodeoxycholic acid (baseline corrected).

Variables	Slope estimates (90% CI) (%)	Acceptance limits for 90% CI^∗^ (%)	Conclusion^∗∗^
*C*_max_ (ng/mL)	41.05 (22.41, 59.70)	(79.69, 120.31)	Not proportional
AUC_0−*t*_ (ng/mL) ∗ (hr)	36.42 (11.71, 61.14)	(79.69, 120.31)	Not proportional
AUC_0−inf_ (ng/mL) ∗ (hr)	36.30 (10.74, 61.86)	(79.69, 120.31)	Not proportional

*Note:* The ratio (*r*) of the highest dose level to the lowest dose level. *C*_max_, maximum plasma concentration; AUC_0−*t*_, area under the plasma concentration versus the time curve from zero time point (before dose) to the last quantifiable concentration; AUC_0−inf_, area under the plasma concentration versus the time curve from zero to infinity.

Abbreviation: CI = confidence interval.

^∗^The lower acceptance limit is calculated as log_*e*_(0.8)/log_*e*_(*r*) + 1. The upper acceptance limit is calculated as log_*e*_(1.25)/log_*e*_(*r*) + 1.

^∗∗^Dose proportionality was concluded if the 90% CI for slope was contained within acceptance limits; otherwise, no dose proportionality was concluded.

**Table 4:** Summary of the treatment emergent adverse event observed with norUrsodeoxycholic acid (safety population).

Adverse events	Cohort 1 500 mg/day (*n* = 14)	Cohort 2 1000 mg/day (*n* = 14)	Cohort 3 1500 mg/day (*n* = 14)
Treatment emergent adverse event, *n* (%)	0	0	1 (7.14)
Increase in the white blood cell count, *n* (%)	0	0	1 (7.14)

## Data Availability

The data used to support the findings of this study are made available upon reasonable request.
